# Psychometric evaluation of the Warwick-Edinburgh Mental Well-being Scale (WEMWBS) with Chinese University Students

**DOI:** 10.1186/s12955-019-1113-1

**Published:** 2019-03-14

**Authors:** Sai-fu Fung

**Affiliations:** Department of Social and Behavioural Sciences, City University of Hong Kong, 83 Tat Chee Avenue, Hong Kong, China

**Keywords:** Chinese students, Validation, Confirmatory factor analysis, WEMWBS, SWEMWBS

## Abstract

**Background:**

The aim of this study was to assess the validity of the Warwick-Edinburgh Mental Well-being Scale (WEMWBS) and the SWEMWBS, the shortened version of the WEMWBS, and conduct a preliminary evaluation of the metric properties of these scales by using a sample of university students in mainland China.

**Methods:**

Nine-hundred and three students from a Chinese university participated in the cross-sectional study. The internal consistency, convergent validity, factorial validity and construct validity of the scales were examined.

**Results:**

The Chinese versions of the WEMWBS and SWEMWBS showed high internal consistency, with Cronbach’s alpha values of 0.930 and 0.884, respectively. The results of the exploratory factor analysis suggested that the 14-item WEMWBS and 7-item SWEMWBS were suitable for a single scale. The WEMWBS and SWEMWBS also showed significant moderate to strong correlations with the other major subjective hedonic and eudemonic scales. Both scales showed good model fit in the confirmatory factor analysis, after reorganising several types of error covariance between the items. However, some items in WEMWBS recorded low validity in the evaluation of internal consistency, convergent validity and factorial validity.

**Conclusions:**

This study demonstrated that the SWEMWBS had high validity, internal consistency and psychometric properties when applied to the sample of Chinese students. Further studies should apply this promising scale to samples of other Chinese populations.

## Background

In recent years, subjective well-being has been recognised as an important theme of study by academics, medical practitioners and international organisations such as the World Health Organization (WHO). Since 2001, the WHO has included mental well-being as one of the main aspects of the definition of health (together with physical and social well-being). Since then, mental well-being has become a prominent factor in the health policies of the UK and many other countries [1–3]. Mental well-being is more than an absence of mental disorder. It is ‘a state of well-being in which the individual realises his or her own abilities, can cope with the normal stresses of life, can work productively and fruitfully, and is able to make a contribution to his or her community’ [4]. Currently, measurements for poor mental health or mental disorders are readily available, but positive mental health is still under-researched, and mental health practitioners lack suitable instruments of measurement [5].

To fill this gap in health-assessment methods, Tennant et al. [5] developed the Warwick-Edinburgh Mental Well-being Scale (WEMWBS). This scale was constructed on the basis of findings from a mixed methods study that included focus group interviews with students and a representative population sample within the UK. The scale uses 14 positively worded items to monitor the mental well-being of the respondents. The WEMWBS assesses two major facets of positive mental health, namely, the hedonic perspective, ‘which focuses on the subjective experience of happiness and life satisfaction’, and the eudaimonic perspective, which focuses on ‘psychological functioning and self realisation’ [5]. The scale was developed with the use of existing instruments, and it aims to ‘capture a wide conception of well-being, including affective-emotional aspects, cognitive-evaluative dimensions and psychological functioning’ [5]. The WEMWBS has been validated on student populations across the UK [6–9].

In the past decade, numerous studies have evaluated the validity of the WEMWBS for different populations. Studies were conducted in the UK to validate the WEMWBS in health care settings and across medically related populations such as veterinary professionals [10], dementia caregivers [11] and users of secondary care mental health services [12]. Maheswaran et al. [13] used the scale as an outcome measure to evaluate mental health interventions on both the individual and group levels. The scale has also been translated into a number of languages and validated in various societies. Studies were conducted on Pakistani health care professionals in the Punjab [14], French psychiatric and general populations [15], Spanish general and student populations [16, 17], the Italian general population [18], Brazilian Portuguese populations [19], Pakistani English-speaking adults in the UK [20] and Norwegian adolescents [21]. Even so, the scale has its critics, and some controversies remain regarding its use. For example, Deary et al. [8] argued that the WEMWBS involves an hierarchy of items, such that males and females tend to get significantly different scores. Recent studies have challenged this claim, with results suggesting that there are no significant differences between the scores of male and female respondents [12]. Following this controversy, the SWEMWBS (a shortened 7-item version of the WEMWBS) was developed to remove potential problems related to gender-biased questions, and this alternative scale was subsequently validated by various studies [8, 9]. In short, the WEMWBS and the SWEMWBS are now widely regarded as some of the few available scales that use positive questions to monitor the mental well-being of the general population. Thus, these scales are generally accepted by medical professionals and practitioners across the UK [11].

This study had two main objectives. First, it aimed to evaluate the psychometric properties of the SWEMWBS. In recent years, various scholars, including the original developers of this scale, i.e., Ruth Tennant and her colleagues, have recommended using the shortened version of the scale, the SWEMWBS [8, 9]. Nevertheless, most of the existing studies on the WEMWBS discuss only the 14-item scale. Thus, the 7-item scale warrants further attention.

Second, the Chinese version of the WEMWBS remains underdeveloped. An official Chinese version of the original WEMWBS was previously validated for use by researchers and medical professionals through tests involving ethnic Chinese people in the UK. [20]. A group of medical doctors in Hong Kong also adapted this Chinese version of the scale to assess patients with mental health disorders [22]. However, it is worth mentioning that these versions of the scale have used traditional Chinese characters, i.e., which are commonly used in Hong Kong and Taiwan, and by Chinese ethnic populations overseas. These characters are significantly different in terms of perception and recognition from the simplified Chinese characters used in mainland China. In particular, the traditional and the simplified Chinese characters have significantly different visual-orthographic and topological properties, which affect their expression and usage [23–25]. Thus, the current Chinese version of the scale may be confusing to users in mainland China, and this difficulty could limit its generalisability and validity within China. Moreover, few studies to date have investigated the application of the Chinese scale among medical-related professionals and patients in a mainland Chinese context. Dong et al. [26] examined the use of the 14-item version in clinical settings among nursing trainees in Wenzhou, China. However, no studies to date have focused on non-medical-related Chinese populations. Thus, it is important to evaluate the scale with other mainland Chinese populations and to further examine the validity of the WEMWBS and SWEMWBS in mainland China.

## Methods

### Participants

The study used a cross-sectional design, with 903 respondents from Huashang College, Guangdong University of Business Studies in mainland China. The sample comprised 792 female and 111 male participants with an average age of 20.56 years (SD = 2.753). This composition of the sample reflected the general demographic characteristics of the university population, as over 80% of the students enrolled in this university were female, according to the official school record.

### Measures

The WEMWBS comprises 14 positively worded questions, designed to evaluate the mental well-being of the respondents. Each question uses a 5-point response scale, ranging from 1 (*none of the time*) to 5 (*all of the time*). The total scores range from 14 to 70 [5].

The 7-item SWEMWBS (using items 1, 2, 3, 6, 7, 9 and 11 from the WEMWBS) is a simplified version of the larger scale. The raw score of the SWEMWBS is calculated on the basis of the developers’ instructions [9]. Prior to this study, the official traditional-Chinese version was only available in the 7-item form, on the official WEMWBS website [27]. This Chinese version of the SWEMWBS was validated in Hong Kong [22].

As in other WEMWBS studies, a rigorous translation process was implemented in this study [15, 17]. A stringent procedure involving several stages was used to translate the official traditional Chinese version of the SWEMWBS into simplified Chinese, and to verify its accuracy. The items not included in the official traditional Chinese version (items 5, 8, 10, 11, 12, 13 and 14) were translated from English into simplified Chinese. Two translators, who were fluent in both English and simplified Chinese, cross-checked the translations, back-translated them into English, and then verified that the meanings of the original English and the translated simplified Chinese versions were identical. Two pilot studies were then conducted, in Xi’an (Northern China) and Guangzhou (Southern China), to ensure that the translated version was free from any cultural biases in the Chinese context. Each pilot study involved five mainland Chinese university students from various academic backgrounds (including accountancy, management, sports sciences, computer sciences and social sciences). Debriefing sessions were conducted, and the pilot participants reported that they had no difficulties understanding or answering the questions in the translated versions. The data from these pilot studies were not included in this study’s data set.

### Procedure

The data were collected from June to July 2018 through the university’s student intranet system. All students were encouraged to participate in a survey related to well-being and quality of life. Participation was completely anonymous and voluntary.

A number of psychometric testing tools and validated instruments were used to analyse the data. Cronbach’s alpha was used to assess the internal consistency of both the WEMWBS and the SWEMWBS [28]. An evaluation on all of the 14 items’ correlation coefficients was conducted, to analyse whether these items warranted scale construction [29].

The evaluation of convergent validity, a sub-type of criterion validity, was used to estimate the correlation coefficients of the WEMWBS and SWEMWBS scores with those of other well-established instruments. The original WEMWBS was cross-checked against the Positive and Negative Affect Scale (PANAS), the Scale of Psychological Well-Being (SPWB), the Short Depression Happiness Scale (SDHS), the WHO Well-being Index (WHO-5), the Satisfaction with Life Scale (SWLS), the Global Life Satisfaction (GLS) scale and the 12-item General Health Questionnaire (GHQ-12) [5]. Other scholars have previously used the WHO-5, the GHQ-12 and the SWLS to evaluate the convergent validity of the WEMWBS [7, 16, 17, 19–21, 26]. To address concerns related to the availability of validated, translated simplified Chinese scales and the length of the questionnaire, the associations between the WEMWBS, the SWEMWBS and the five major subjective hedonic and eudemonic scales related to general health, psychological functioning and life satisfaction were all examined. The participants were asked to complete a questionnaire with 55 items, which comprised the original 14-item WEMWBS [5], the WHO-5 [30–32], the GHQ-12 [33], the SWLS [34–37], the Subjective Happiness Scale (SHS) [38], the Personal Well-being Index (PWI) [39] and several basic demographic questions.

Exploratory factor analysis was conducted, with principal component analysis used to evaluate the single factor model suggested in the literature [5, 9, 26, 40]. In this analysis, an item with a factor loading over 0.50 could be interpreted as having practical significance when the sample size was more than 350 [29].

Confirmatory factor analysis was used to evaluate the scales’ construct validities [41–43]. Diagonally weighted least squares (DWLS) estimation method was used due to the WEMWBS and SWEMWBS with ordinal nature that constructed by five point Likert scale items. The DWLS was regarded as less biased, work well for items with different categories (ranging from two to seven), more optimal fit, and work better in larger sample size for ordinal variables [44–48]. The model fit and cut-off criteria were evaluated on the basis of the cut-off values indicated in the existing structural equation modelling (SEM) literature. According to these criteria, a comparative fit index (CFI) of over 0.950, a Tucker-Lewis fit index (TLI) of over 0.950, a standardised root mean square residual (SRMR) under 0.08 and an root mean square error of approximation (RMSEA) under 0.06, were considered acceptable [12, 29, 49–51]. In addition, to evaluate the results from large samples, an acceptable model could be indicated by χ^2^/df ≤ 3 [52, 53]. All of these analyses were implemented with the IBM SPSS 25.0 and the lavaan package version 0.6–3 [54] in R version 3.5.2 software.

## Results

There were 5568 viewers who clicked on the online survey webpage through the school intranet system, and a total of 903 participants completed the questionnaire via their own desktop computers (0.23%) or their smartphone (99.67%) web browsers. Each participant was able to submit the survey only once. Most of the students completed the self-administered questionnaire within 10 min.

### Internal consistency

The means, standardised deviations, skewness, kurtosis and corrected item-total correlations for all 14 items of the WEMWBS (*N* = 903) are presented in Table 1. The corrected item-to-total correlations for the 14-item WEMWBS ranged from 0.385 to 0.786. It is worth noting that item 4 received the lowest value (0.385), and this finding required further attention in the subsequent tests. The Spearman correlation matrix table, showing the relations between all of the 14 items, is presented in the Appendix. All of the correlation coefficients (except item 4) were over 0.300, which was appropriate for the factor analysis [29, 55]. The Cronbach’s alphas of the WEMWBS and the SWEMWBS were 0.930 and 0.884, respectively, which were both similar to the original WEMWBS Cronbach’s alpha value (0.890) and to the values reported in other related studies [5, 6, 14, 20]. The correlation between the WEMWBS and SWEMWBS was 0.950 (*p* < 0.001), which corresponded to the value of 0.954 reported by the original developers [9]. No significant differences between the male and female respondents were observed in the scale scores, according to the independent-sample *t*-test results.

**Table 1:** Descriptive statistics for the WEMWBS and SWEMWBS^#^ items

Item	Mean	SD	Skewness	Kurtosis	Corrected item-total correlations	Cronbach's alpha, if item deleted
WEMWBS1^#^	3.44	0.932	−0.171	− 0.348	0.671	0.924
WEMWBS2^#^	3.47	0.935	−0.162	−0.336	0.720	0.923
WEMWBS3^#^	3.14	0.879	0.154	−0.269	0.655	0.925
WEMWBS4	3.24	0.883	0.032	−0.096	0.385	0.933
WEMWBS5	3.27	0.849	0.112	−0.209	0.708	0.923
WEMWBS6^#^	3.25	0.795	0.100	0.201	0.710	0.923
WEMWBS7^#^	3.34	0.806	0.083	−0.121	0.684	0.924
WEMWBS8	3.43	0.839	−0.051	−0.129	0.786	0.921
WEMWBS9^#^	3.35	0.921	−0.014	−0.403	0.700	0.923
WEMWBS10	3.22	0.920	0.054	−0.313	0.770	0.921
WEMWBS11^#^	3.34	0.870	0.077	−0.337	0.704	0.923
WEMWBS12	3.56	0.996	−0.157	−0.670	0.606	0.927
WEMWBS13	3.80	0.862	−0.136	−0.693	0.565	0.928
WEMWBS14	3.57	0.849	−0.079	−0.135	0.754	0.922

Data captured with # is related to SWEMWBS

### Convergent validity

In previous studies, the WEMWBS was reported to have significant moderate to high positive correlations with indictors related to well-being, positive affect, life satisfaction and overall health [5, 7, 16, 17, 19–21, 26]. The results in this study replicated those found in the above-listed prior studies. Table 2 reports the correlations between the overall WEMWBS and the SWEMWBS scales, the specific items and the other construct-related scales. Significant and strongly positive correlations were observed, with WHO-5 (*r* = 0.499, *p* < 0.001), SHS (*r* = 0.584, *p* < 0.001), SWLS (*r* = 0.593, *p* < 0.001) and PWI (*r* = 0.674, *p* < 0.001). However, items 4 and 13 showed only small to moderate associations with the above-listed scales (Table 3).

**Table 2:** Correlations between the WEMWBS and SWEMWBS in relation to other construct-related scales

Scale	WEMWBS	SWEMWBS
WHO-5	0.499	0.438
GHQ-12	−0.515	− 0.477
SWLS	0.593	0.570
SHS	0.584	0.547
PWI	0.674	0.652

Note: All correlations are significant at the 0.001 level (2-tailed)

**Table 3:** Correlations between the WEMWBS and SWEMWBS^#^ items and the other construct-related scales

Item	WHO-5	GHQ-12	SHS	SWLS	PWI
WEMWBS1^#^	0.356	−0.424	0.520	0.485	0.534
WEMWBS2^#^	0.359	−0.442	0.472	0.459	0.535
WEMWBS3^#^	0.391	−0.376	0.502	0.521	0.533
WEMWBS4	0.180	−0.109	0.143	0.223	0.227
WEMWBS5	0.393	−0.357	0.440	0.465	0.510
WEMWBS6^#^	0.315	−0.399	0.400	0.426	0.504
WEMWBS7^#^	0.322	−0.375	0.384	0.425	0.502
WEMWBS8	0.376	−0.449	0.498	0.514	0.567
WEMWBS9^#^	0.353	−0.389	0.447	0.418	0.520
WEMWBS10	0.373	−0.432	0.491	0.496	0.558
WEMWBS11^#^	0.282	−0.400	0.384	0.397	0.484
WEMWBS12	0.299	−0.334	0.396	0.422	0.457
WEMWBS13	0.176	−0.279	0.282	0.252	0.332
WEMWBS14	0.367	−0.450	0.543	0.499	0.565

Note: All correlations are significant at the 0.001 level (2-tailed)

Data captured with # is related to SWEMWBS

Previous research also showed that the WEMWBS was significantly and negatively correlated with symptoms of anxiety and depression [5, 7, 11, 17]. The results from this study (Table 2) replicated those findings. The two tested scales had strongly negative correlations with GHQ-12 (*r* = − 0.515, *p* < 0.001), but items 4 and 13 showed small to moderate correlation coefficients (Table 3). In short, the Chinese versions of the WEMWBS and the SWEMWBS showed good criterion validity with the other construct-related measures.

### Factorial validity

The factor analysis results showed Kaiser-Mayer-Olkin (KMO) values of 0.946 for the WEMWBS and 0.882 for the SWEMWBS. The results of the exploratory factor analysis (Table 4) suggested that one extracted component from the 14-item WEMWBS explained 53.3% of the variance, with factor loadings from 0.187 to 0.691. Among all of the other items, items 4, 12 and 13 reported low pattern coefficients, with Factor 1 yielding 0.187, 0.434 and 0.376 respectively. The 7-item SWEMWBS also extracted one factor that contributed 59.3% of the variance, with factor loadings from 0.537 to 0.650. This set of findings was aligned with the results of other validation studies [15, 19]. These results suggested that the SWEMWBS had better factorial validity than the original full version.

**Table 4:** Factor loading results from exploratory factor analysis (using principal component analysis)

WEMWBS	Factor 1	SWEMWBS	Factor 1
Item 1	0.523	Item 1	0.564
Item 2	0.593	Item 2	0.633
Item 3	0.504	Item 3	0.537
Item 4	0.187		
Item 5	0.572		
Item 6	0.585	Item 6	0.650
Item 7	0.552	Item 7	0.623
Item 8	0.691		
Item 9	0.562	Item 9	0.538
Item 10	0.671		
Item 11	0.580	Item 11	0.608
Item 12	0.434		
Item 13	0.376		
Item 14	0.634		

### Construct validity

Table 5 shows the results of the confirmatory factor analysis on the 14-item WEMWBS and the 7-item SWEMWBS. Model 1 evaluated the full scale of the WEMWBS without correlations among the measurement errors. The results of Model 1 suggested that the scale did not fit the model well, as χ^2^ (805.373) = 77, *p* < 0.001, SRMR = 0.056, CFI = 0.990, TLI = 0.989 and RMSEA = 0.102. Model 2 included various covariance factors between the error terms for items 1 to 7, and 10 to 14. The results demonstrated good model fit, with χ^2^ (188.076) / 67 = 2.81, *p* < 0.001, SRMR = 0.025, CFI = 0.998, TLI = 0.998 and RMSEA = 0.045.

**Table 5:** Confirmatory factor analysis of the WEMWBS

Model	χ^2^	df	RMSEA	CFI	TLI	SRMR
WEMWBS						
1	805.373***	77	.102	.990	.989	.056
2^a^	188.076***	67	.045	.998	.998	.031
SWEMWBS						
3	310.356***	14	.153	.986	.979	.067
4^b^	17.452*	9	.032	.999	.999	.017

^a^ Includes the covariance between the error terms for items WEMWBS1 and WEMWBS2, WEMWBS1 and WEMWBS3, WEMWBS4 and WEMWBS5, WEMWBS4 and WEMWBS13, WEMWBS6 and WEMWBS7, WEMWBS6 and WEMWBS11, WEMWBS7 and WEMWBS11, WEMWBS12 and WEMWBS13, WEMWBS12 and WEMWBS14, and WEMWBS13, and WEMWBS14

^b^ Includes the covariance between the error terms for items WEMWBS1 and WEMWBS2, WEMWBS1 and WEMWBS3, WEMWBS2 and WEMWBS3, WEMWBS6 and WEMWBS9, and WEMWBS7 and WEMWBS9

* *p* < .05. *** *p* < .001

**Fig. 1:**
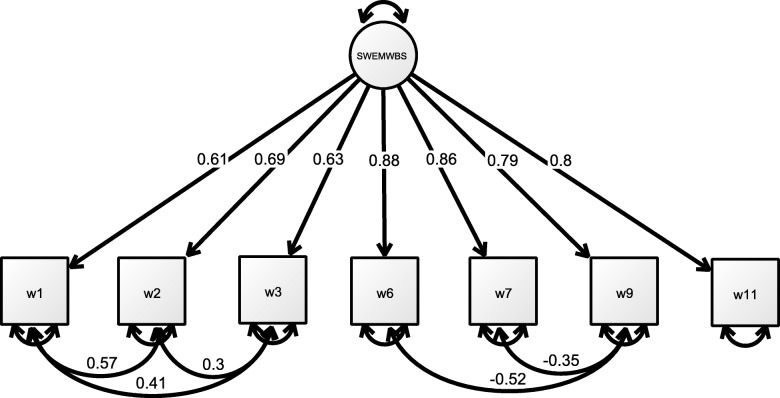
Final standardised model of the 7-item SWEMWBS

Model 3 provide the CFA analysis of the shortened version of the 7-item scale, without correlating the error terms. The results suggested that this scale did not fulfil the cut-off values, as χ^2^ (310.356) = 14, *p* < 0.001, SRMR = 0.067, CFI = 0.986, TLI = 0.979 and RMSEA = 0.153. Model 4 evaluated the SWEMWBS, with the error correlations based on the modification indices. The data suggested that the SWEMWBS was suitable for a single scale. The results indicated good model fit, as χ^2^ (17.452)/9 = 1.94, *p* = 0.042, SRMR = 0.017, CFI = 0.999, TLI = 0.999 and RMSEA = 0.032. The covariance between the error terms is presented in Fig. 1. Overall, the results indicated that both the WEMWBS and SWEMWBS had a generally good fit for one underlying factor with post hoc modification.

## Discussion

This study has aimed to test and validate the WEMWBS [5] and the SWEMWBS [9] by using a sample of Chinese university students. The analyses of internal consistency suggested that both scales produced results that were similarly robust as those from the original and the other translated versions of the scale, such as the Portuguese, Spanish, Italian, Urdu and Norwegian versions [14–16, 18–21]. The mean scores for the WEMWBS and SWEMWBS were 47.41 (SD = 8.93) and 21.46 (SD = 4.08), respectively, which were similar to the scores reported in other recent studies [7, 10, 14, 15, 20].

However, the results also showed that the SWEMWBS had better internal consistency, convergent validity, and factorial validity than the 14-item of WEMWBS. In particular, items 4, 12 and 13 in the WEMWBS exhibited relatively low values in at least one of the following analyses: correlated item-total correlations, correlations with other WEMWBS items, correlations with other construct-related scales, and factor loadings in exploratory factor analysis. Hence, various scholars have recommended using the shortened version, which covers the major factors related to psychological, hedonic and eudemonic well-being, and is a useful tool for studying the populations concerned [8, 9]. Nonetheless, the proposers of the SWEMWBS still suggested that because the ‘SWEMWBS is embedded within the larger WEMWBS, it may be appropriate to continue to collect data on the full 14 items’ [9].

The results of this study showed that the Chinese-translated versions of the SWEMWBS are useful and reliable tools for studying the mental well-being of participants in a Chinese university setting. Several previous studies have validated and investigated the use of these scales among Chinese medical professionals and mental patients in Hong Kong [22, 26]. Given that the original English scale was based on research involving student populations in England and Scotland [5], this study attempted to validate the Chinese translated scale with a similar population, i.e., university students. The results demonstrated that the translated simplified Chinese version of the SWEMWBS was comparable with the original English version.

Mental well-being has emerged as a major factor in assessments of psychological well-being, and this aspect of health is attracting increasing attention from both the Chinese government and from relevant authorities such as the National Health and Family Planning Commission. In 2017, the Chinese government issued its first guidelines for improving mental health in schools, workplaces and hospitals [2]. The validated SWEMWBS can provide a handy and useful psychometric tool for scholars, medical professionals and other major stakeholders to use for examining the current state of mental well-being in mainland China.

This study used innovative and effective data-collection methods. The data-collection process involved a smartphone social network site (SNS) app delivered via the university’s student intranet. Using this type of information and communication technology (ICT) provided at least three advantages over using traditional data-collection methods. First, this method significantly reduced the research costs, because the study did not rely on research assistants to distribute and collect the questionnaires. Second, it greatly reduced the amount of time required for data entry, and it avoided input mistakes by the researcher. Third, this data-collection method was environmentally friendly, because it did not involve printing questionnaires, which saved at least 10,000 pieces of paper.

This study has several potential limitations. The first is related to the usage of correlated errors in the confirmatory factor analysis. The results for the WEMWBS and SWEMWBS as presented in Model 2 and 4 were indicated as good model fit. Without the correlation of errors, the scales would fail to pass the cut-off criteria for model fit. This situation is not uncommon, as many scholars support the practice of changing the model to improve the fit [56–59]. Indeed, the original scale developers [5], the validation studies on a Spanish population [17], on students from England and Scotland [7] and on Norwegian adolescents [21] all reported covariance in the error terms based on modification indices. This study has presented both the pre- and post-correlation fit values for interpreting the results, to enable the readers to better comprehend the differences involved. In short, after reorganising several types of error covariance between the items in the WEMWBS (Model 2) and SWEMWBS (Model 4), the CFA results fulfilled all of the stringent cut-off criteria for a good model fit.

Another limitation of this study is the failure to provide an accurate response rate, due to a technical constraint. The school intranet system only reported the total number of viewers who clicked on the questionnaire page, but did not keep track of the viewers’ identities. Although the system allowed each user to submit only one questionnaire, it was possible that some users clicked on the webpage multiple times before making their final submissions. This limitation, however, was compensated to some degree by larger sample size that this data-collection method enabled. Future online surveys via school intranet systems may find ways to address this response rate problem. However, in doing so, the researchers also need to avoid any infringement on the privacy and anonymity of the respondents.

Last, this study did not include comparisons with the PANAS, SPWB, SDHS or GLS, as Tennant et al. [5] were able to do. These scales were omitted due to the unavailability of reliable Chinese translated versions, and due to concerns that an overly lengthy questionnaire could discourage students from participating in the study. To overcome this limitation, this study conducted comparisons with the WHO-5, GHQ-12, SWLS, SHS and PWI scales, which have also been commonly used in other studies to evaluate WEMWBS criterion validity [7, 16, 17, 19–21, 26].

## Conclusions

The results of this study showed that the 7-item SWEMWBS was more appropriate for use in a sample of Chinese university students than the 14-item version of WEMWBS. The SWEMWBS was found to be a valid tool for examining the mental well-being of the sample in this study, as this scale showed better internal consistency, convergence validity and factorial validity than the WEMWBS. These results also shed light on the research agenda for future studies, suggesting the following research goals: 1) to examine the validity of this new and promising instrument in populations with more diverse backgrounds, including different age groups and other professional populations in China; 2) to use a pretest-posttest design on Chinese mental well-being intervention programmes to further evaluate the scale’s effectiveness; and 3) to conduct explorative studies for further analysing the variables that are related to positive mental health in the context of mainland China.

## Appendix

**Table 6:** Correlation matrix for the 14-item WEMWBS and the 7-item SWEMWBS ^#^

Item	1	2	3	4	5	6	7	8	9	10	11	12	13	14
WEMWBS1#	1.000													
WEMWBS2^#^	0.688	1.000												
WEMWBS3^#^	0.544	0.525	1.000											
WEMWBS4	0.267	0.283	0.288	1.000										
WEMWBS5	0.525	0.536	0.519	0.364	1.000									
WEMWBS6^#^	0.449	0.515	0.472	0.316	0.555	1.000								
WEMWBS7^#^	0.402	0.495	0.443	0.242	0.519	0.678	1.000							
WEMWBS8	0.542	0.610	0.551	0.304	0.555	0.591	0.586	1.000						
WEMWBS9^#^	0.457	0.502	0.429	0.306	0.508	0.471	0.505	0.585	1.000					
WEMWBS10	0.526	0.600	0.506	0.271	0.550	0.590	0.537	0.696	0.614	1.000				
WEMWBS11^#^	0.433	0.513	0.406	0.207	0.516	0.595	0.616	0.587	0.552	0.643	1.000			
WEMWBS12	0.441	0.472	0.410	0.236	0.385	0.395	0.378	0.492	0.498	0.500	0.461	1.000		
WEMWBS13	0.365	0.407	0.264	0.319	0.397	0.345	0.377	0.421	0.473	0.399	0.459	0.522	1.000	
WEMWBS14	0.538	0.567	0.530	0.285	0.527	0.482	0.515	0.626	0.588	0.596	0.542	0.602	0.601	1.000

Note: All correlations are significant at the 0.001 level (2-tailed)

Data captured with # is related to SWEMWBS

## Abbreviations

CFI: Comparative fit index
GHQ-12: 12-item General Health Questionnaire
GLS: Global Life Satisfaction
ICT: Information and communication technology
PANAS: Positive and Negative Affect Scale
PWI: Personal Well-being Index
RMSEA: Root mean square error of approximation
SD: Standardised deviation
SDHS: Short Depression Happiness Scale
SEM: Structural equation modelling
SHS: Subjective Happiness Scale
SNS: Social network site
SPWB: Scale of Psychological Well-Being
SRMR: Standardised root mean square residual
SWEMWBS: Shortened version of the Warwick-Edinburgh Mental Well-being Scale
SWLS: Satisfaction with Life Scale
TLI: Tucker-Lewis fit index
WEMWBS: Warwick-Edinburgh Mental Well-being Scale
WHO: World Health Organization
WHO-5: WHO Well-being Index
